# Two *N*-{[4-(3-aryl-4-sydnonyl­idene­amino)-5-sulfan­yl­idene-1*H*-1,2,4-triazol-3-yl]meth­yl}benzamides as disordered ethanol monosolvates

**DOI:** 10.1107/S2056989020007483

**Published:** 2020-06-09

**Authors:** Chayanna Harish Chinthal, Hemmige S. Yathirajan, Anish Kumar Kadambar, Balakrishna Kalluraya, Sabine Foro, Christopher Glidewell

**Affiliations:** a Department of Studies in Chemistry, University of Mysore, Manasagangotri, Mysuru-570 006, India; b Department of Studies in Chemistry, Mangalore University, Mangalagangotri, Mangalore-574199, India; c Institute of Materials Science, Darmstadt University of Technology, Alarich-Weiss-Strasse 2, D-64287 Darmstadt, Germany; d School of Chemistry, University of St Andrews, St Andrews, Fife KY16 9ST, UK

**Keywords:** synthesis, heterocyclic compounds, sydnones, 1,2,4-triazoles, crystal structure, disorder, hydrogen bonding, supra­molecular assembly

## Abstract

Two new *N*-{[4-(3-aryl-4-sydnonyl­idene­amino)-5-sulfanyl­idene-1*H*-1,2,4-triazol-3-yl]meth­yl}benzamides have been prepared by acid-promoted condensation reactions between 3-aryl-4-formyl­sydnones and *N*-[(4-amino-5-sulfanyl­idene-1*H*-1,2,4-triazol-3-yl)meth­yl]benzamide, and both have been crystallized as ethanol monosolvates. *N*-{[4-(3-Phenyl-4-sydnonyl­idene­amino)-5-sulfanyl­idene-1*H*-1,2,4-triazol-3-yl]meth­yl}benzamide ethanol monosolvate, C_19_H_15_N_7_O_3_S·C_2_H_6_O (**I**), and *N*-({4-[3-(4-methyl­phen­yl)-4-sydnonyl­idene­amino]-5-sulfanyl­idene-1*H*-1,2,4-triazol-3-yl}meth­yl)benzamide ethanol monosolvate, C_20_H_17_N_7_O_3_S·C_2_H_6_O (**II**), differ only in the presence of a methyl group for (**II**) instead of a hydrogen atom for (**I**), and in both of them the ethanol component is disordered over two sets of atomic sites having occupancies of 0.836 (6) and 0.164 (6) in (**I**), and 0.906 (6) and 0.094 (6) in (**II**). Combinations of O—H⋯O and N—H⋯O hydrogen bonds link the mol­ecules into cyclic, centrosymmetric four-mol­ecule aggregates. Comparisons are made with the structures of some related compounds.

## Abstract 2

In each of the title newly synthesized and closely related *N*-{[4-(3-aryl-4-sydnonyl­idene­amino)-5-sulfanyl­idene-1*H*-1,2,4-triazol-3-yl]meth­yl}benzamides, which crystallized as ethanol monosolvates, the independent components are linked by hydrogen bonds to form centrosymmetric four-mol­ecule aggregates.

## Chemical context   

Compounds containing the sydnone [= 1,2,3-oxa­diazol-5(2*H*)-one] system have been shown to exhibit a wide range of biological activities, including analgesic (Kalluraya *et al.*, 2001 ▸, 2002 ▸), and both anti­helminthic and anti-inflammatory properties (Kalluraya *et al.*, 2001 ▸). In addition, compounds that combine sydnone units with other heterocyclic units such as thia­zoles (Kalluraya *et al.*, 2001 ▸) or 1,2,4-triazines (Hegde *et al.*, 2008 ▸), have been shown to exhibit CNS depressant and anti­microbial activities. Seeking to continue our studies in this area, we have now developed a synthesis of analogous compounds containing 3-aryl­sydnone and 1,2,4-triazole moieties.

**Figure 1:**
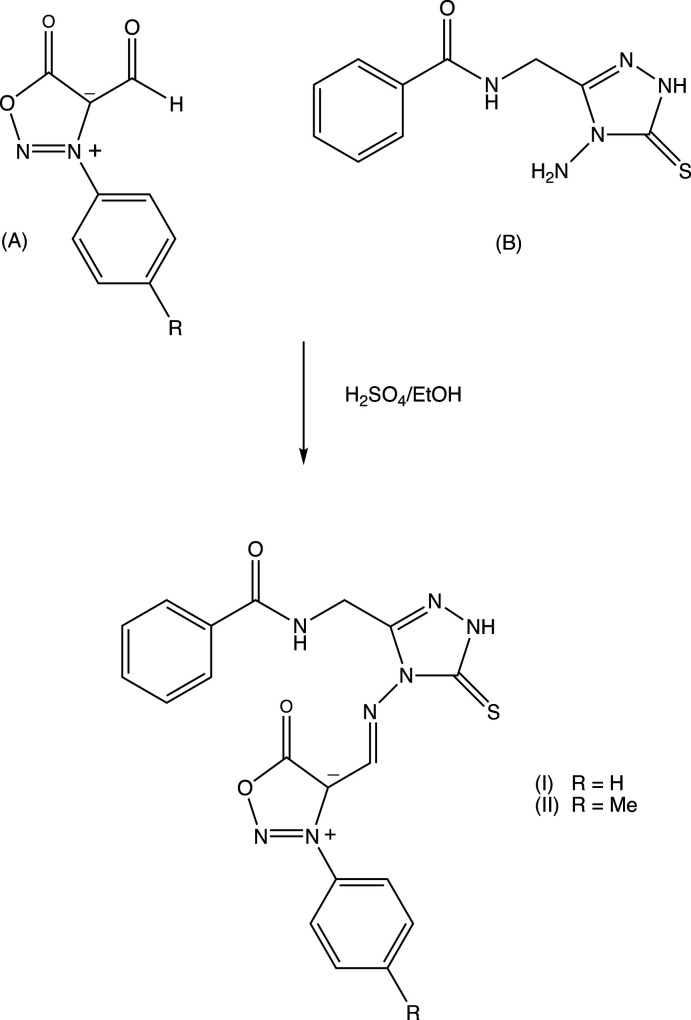
The reaction sequence leading to the formation of compounds (**I**) and (**II**).

We report here the syntheses and mol­ecular and supra­molecular structures of two closely related compounds, namely *N*-{[4-(3-phenyl-4-sydnonyl­idene­amino)-5-sulfanyl­idene-1*H*-1,2,4-triazol-3-yl]meth­yl}benzamide (**I**) and *N*-({4-[3-(4-methyl­phen­yl)-4-sydnonyl­idene­amino]-5-sulfanyl­idene-1*H*-1,2,4-triazol-3-yl}meth­yl)benzamide (**II**). Compounds (**I**) and (**II**) were prepared using an acid-mediated condensation between the 3-aryl-4-formyl­sydnones (A) (Fig. 1 ▸) and the 4-amino­triazole derivative (B). The sydnone inter­mediates (A) had themselves been prepared by cyclo­dehydration of the corresponding *N*-aryl-*N*-nitro­soplycines followed by Vilsmaier–Haack formyl­ation (Goh *et al.*, 2010 ▸), while the inter­mediate (B) was prepared by the fusion-induced condensation of *N*-benzoyl­glycine with thio­carbohydrazide, S=C(NHNH_2_)_2_ (Kalluraya *et al.*, 2007 ▸).
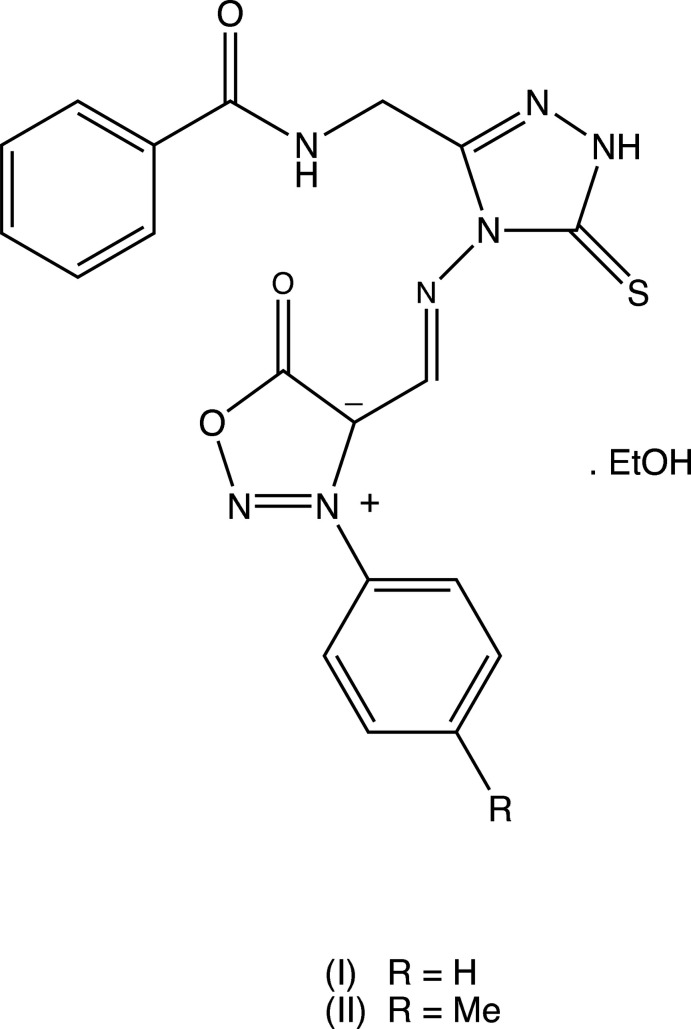


## Structural commentary   

**Figure 2:**
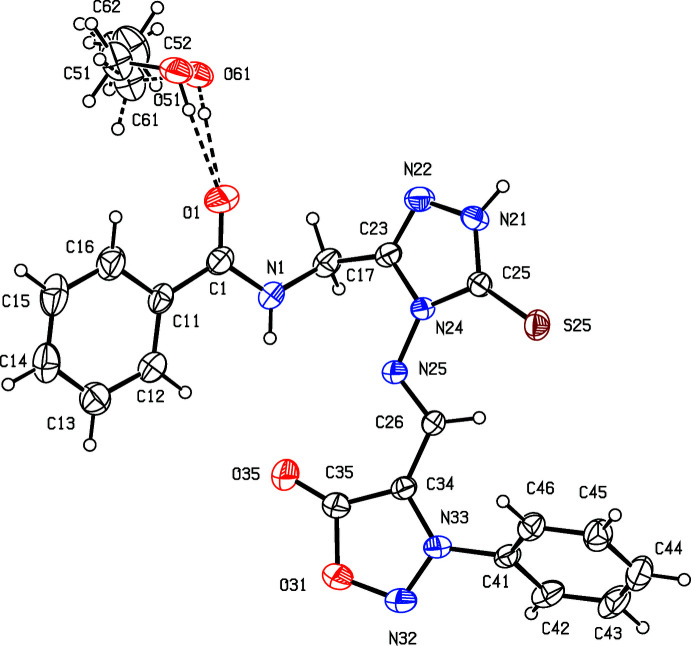
The independent mol­ecular components of compound (**I**), showing the atom-labelling scheme, the disorder of the ethanol component, and the hydrogen bonds, drawn as dashed lines, within the asymmetric unit. Displacement ellipsoids are drawn at the 30% probability level; the major disorder component of the ethanol is drawn with full lines, and the minor component is drawn with broken lines.

The mol­ecular and crystal structures of compounds (**I**) and (**II)** are closely related and differ only in the methyl group that is attached to C44 for (**II**) instead of a hydrogen atom for (**I**): each structure can readily be refined starting from the atomic coordinates of the other, provided that the necessary adjustment is made to the substituent at atom C44 (Figs. 2 ▸ and 3 ▸). Both compounds crystallized from ethanol/DMF as ethanol monosolvates, and in each structure the ethanol component is disordered over two sets of atomic sites, having occupancies of 0.836 (6) and 0.164 (6) in (**I**), and 0.906 (6) and 0.094 (6) in (**II**).

The triazole ring is present in both structures in the 1,2,4-triazol-5(4*H*)-thione form, as shown by the localization of the H atom on N21 in a difference-Fourier map and the subsequent refinement of its atomic coordinates, by the inter­molecular hydrogen bonds (Tables 1 ▸ and 2 ▸), and by the C—S distances, 1.6657 (18) Å in (**I**) and 1.661 (3) Å in (**II**). These values are typical for those found in thio­nes [mean value 1.671 Å; Allen *et al.*, 1987 ▸], and they are far shorter than those found in aromatic thiols and thio­ethers (mean value 1.771 Å).

Within the sydnone rings, the N—N distances have values typical of double bonds, *viz*. 1.300 (2) Å in (**I**) and 1.299 (3) Å in (**II**), while the exocyclic C=O distance in each structure is shorter than that for the amidic carbonyl unit.

Of the four independent aromatic rings within the mol­ecules of (**I**) and (**II**), no two are co-planar or even parallel, so that the mol­ecules exhibit no inter­nal symmetry and hence are conformationally chiral. Although there is a short intra­molecular N1—H1⋯N25 contact in both (**I**) and (**II**) (Tables 1 ▸ and 2 ▸), the resulting rings are non-planar, but instead adopt an envelope conformation, folded across the line N1⋯C23.

## Supra­molecular features   

**Figure 4:**
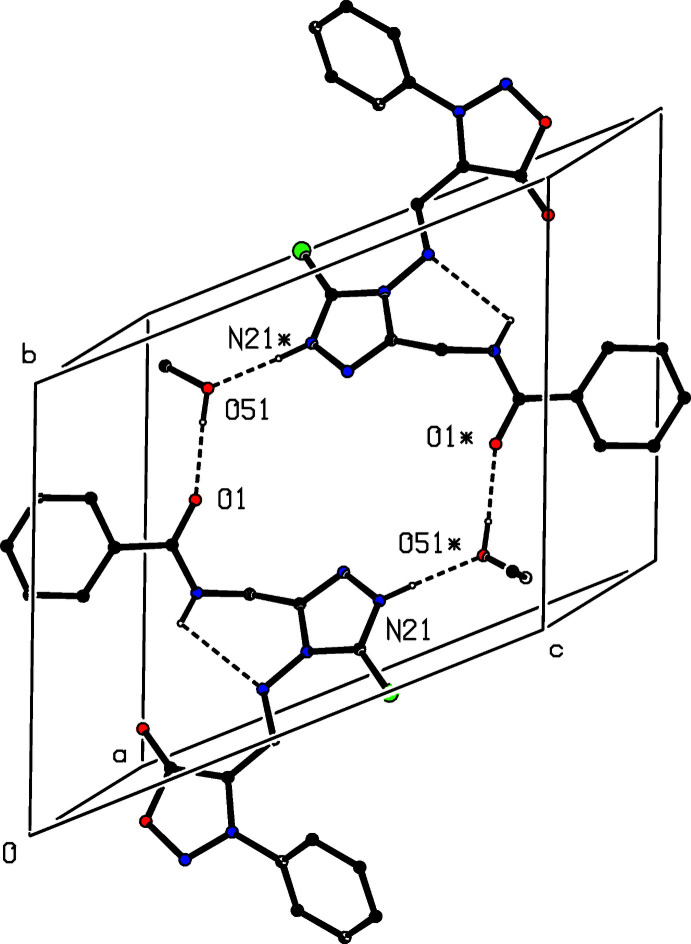
Part of the crystal structure of compound (**I**) showing the formation of a hydrogen-bonded four-mol­ecule aggregate. Hydrogen bonds are drawn as dashed lines and, for the sake of clarity, the minor disorder component of the ethanol mol­ecules and the H atoms bonded to C atom have been omitted. The atoms marked with an asterisk (*) are at the symmetry position (1 − *x*, 1 − *y*, 1 − *z*).

The supra­molecular assemblies in the crystal structures of (**I**) and (**II**) are almost identical and very simple. Within the asymmetric unit of each structure (Figs. 2 ▸ and 3 ▸), the ethanol solvent mol­ecule is linked to the amide unit *via* O51—H51⋯O1 hydrogen bonds. Inversion-related pairs of these units are linked by N—H⋯O hydrogen bonds to form a cyclic centrosymmetric four-mol­ecular aggregate [shown only for (**I**) in Fig. 4 ▸] containing an 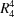
(2) motif (Etter, 1990 ▸; Etter *et al.*, 1990 ▸; Bernstein *et al.*, 1995 ▸). The same motif occurs in the crystal structure of compound (**II**), and there are no significant direction-specific inter­actions between these aggregates.

## Database survey   

It is of inter­est briefly to compare the structures of compounds (**I**) and (**II**) with those of some related compounds. In the structure of 4-amino-3-(1,2,4-triazol-1-yl)-1*H*-1,2,4-triazole-5(4*H*)-thione (**III**), three independent N—H⋯N hydrogen bonds link the mol­ecules into a three-dimensional network structure (Xu *et al.*, 2005*a* ▸). By contrast, in each of 5-[(4-phenyl-1*H*-1,2,3-triazol-1-yl)meth­yl]-1,3,4-oxa­diazole-2-thione (**IV**) (Zhang *et al.*, 2006*a* ▸) and 5-{[4-(4-meth­oxy­phen­yl)-1*H*-1,2,3-triazol-1-yl]meth­yl}-1,3,4-oxa­diazole-2-thione (**V**) (Zhang *et al.*, 2006*b* ▸), a single N—H⋯N hydrogen bond links mol­ecules that are related by translation into *C*(8) chains, running parallel to [001] and [010], respectively, in the triclinic unit cells. Although no crystal structure has yet been reported for the inter­mediate (B) (Fig. 1 ▸) used in the synthesis of compounds (**I**) and (**II**), the fact that all of compounds (**I**)–(**V**) crystallize in the thione form makes it seem likely that the inter­mediate also exists in this tautomeric form in the solid state, although it may well exist as an equilibrium mixture of thione and thiol (mercapto) forms in solution, with the position of equilibrium possibly differing from one solvent to another. However, it must be emphasized that, to date, no studies have been made of the constitution of this inter­mediate in solution. On the other hand, a masked form of the thiol tautomer is present in 2-{5-[(1*H*-1,2,4-triazol-1-yl)meth­yl]-1,3,4-oxa­diazol-2-yl­thio}-1-(2,4-di­chloro­phen­yl)ethanone (**VI**) (Xu *et al.*, 2005*b* ▸), where mol­ecules which are related by a 2_1_ screw axis are linked by a single C—H⋯N hydrogen bond to form *C*(14) chains.

## Synthesis and crystallization   

Previously published methods were used for the preparation of the 3-aryl-4-formyl­sydnones (A) (Fig. 1 ▸) (Goh *et al.*, 2010 ▸) and *N*-[(4-amino-5-sulfanyl­idene-1*H*-1,2,4-triazol3-yl)meth­yl]benzamide (B) (Kalluraya *et al.*, 2007 ▸). For the preparation of compounds (**I**) and (**II**), the appropriate inter­mediate (A) [4.6 mmol; 870 mg for (**I**) or 940 mg for (**II**)] was added to a solution of (B) (4.6 mmol, 1.00 g) in ethanol (15 ml). Concentrated sulfuric acid (0.5 ml) was then added to each of these mixtures, under vigorous stirring, and stirring was then continued for 4 h. The resulting solid products were collected by filtration and then washed, first with ethanol and then with water, before being dried in air. Compound (**I**), yield 72%, m. p. 435 K, IR (cm^−1^) 3170 (NH), 1740 (C=O), 1660 (C=O), 1590 (C=N). Compound (**II**), yield 76%, m. p. 505 K, IR (cm^−1^) 3149 (NH), 1769 (C=O), 1665 (C=O), 1595 (C=N). Crystals of (**I**) and (**II**) suitable for single-crystal X-ray diffraction were grown by slow evaporation, at ambient temperature and in the presence of air, of solutions in ethanol/*N*,*N*-di­methyl­formamide mixtures (initial composition 7:3, *v*/*v*).

## Refinement   

Crystal data, data collection and refinement details are summarized in Table 3 ▸. In both compounds, the ethanol component is disordered over two sets of atomic sites having unequal occupancies: for the minor disorder components, the bond distances and the 1,3 (non-bonded) distances were restrained to be the same as the corresponding distances in the major disorder components, subjected to s.u. values of 0.01 and 0.02 Å, respectively. In addition, the anisotropic dis­place­ment parameters for corresponding pairs of partial-occupancy atoms occupying essentially the same physical space were constrained to be the same. All H atoms, apart from those in the minor disorder components, were located in difference-Fourier maps. The H atoms bonded to C atoms were then treated as riding atoms in geometrically idealized positions with C—H distances of 0.93 Å (alkenyl and aromatic), 0.96 Å (CH_3_) or 0.97 Å (CH_2_), and with *U*_iso_(H) = *kU*_eq_(C), where *k* = 1.5 for the methyl groups, which were allowed to rotate but not to tilt, and 1.2 for all other H atoms bonded to C atoms: the H atoms bonded to C atoms in the minor disorder components were included on the same basis. For the H atoms bonded to N atoms, the atomic coordinates were refined with *U*_iso_(H) = 1.2*U*_eq_(N), giving the N—H distances shown in Tables 1 ▸ and 2 ▸. For the major disorder components of the ethanol mol­ecules, the H atoms bonded to O atoms were treated as riding atoms with O—H distances of 0.82 Å and with 1.5*U*_eq_(O). However, using the normal riding models for hydroxyl H atoms, it was not possible to establish satisfactory positions for these H atoms in the minor disorder components, and accordingly they were included in calculated positions, riding at 0.82 Å from the atoms O61, at positions calculated by inter­polation along the O61⋯O1 vectors, again with *U*_iso_(H) = 1.5*U*_eq_(O). The refined occupancies for the disorder components were 0.836 (6) and 0.164 (6) in (**I**), and 0.906 (6) and 0.094 (6) in (**II**).

## Supplementary Material

Crystal structure: contains datablock(s) global, I, II. DOI: 10.1107/S2056989020007483/wm5563sup1.cif

Structure factors: contains datablock(s) I. DOI: 10.1107/S2056989020007483/wm5563Isup2.hkl

Structure factors: contains datablock(s) II. DOI: 10.1107/S2056989020007483/wm5563IIsup3.hkl

CCDC references: 2007902, 2007901

Additional supporting information:  crystallographic information; 3D view; checkCIF report

## Figures and Tables

**Figure 3:**
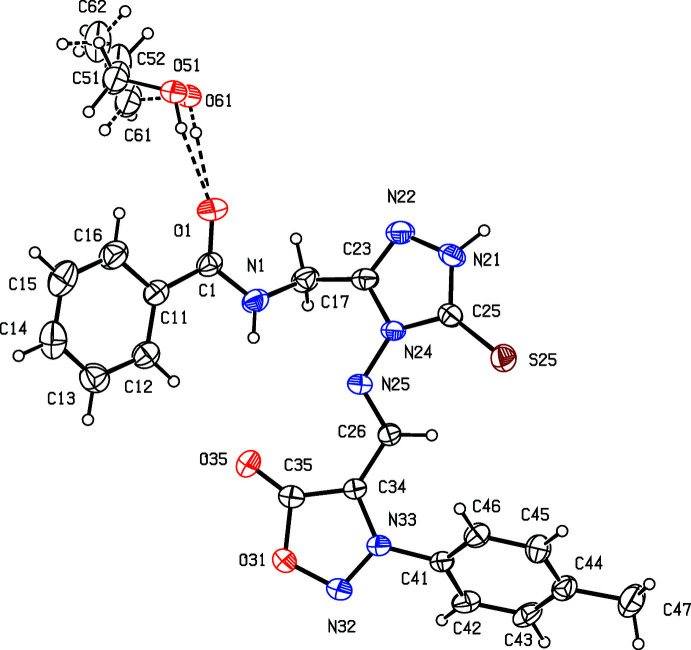
The independent mol­ecular components of compound (**II**), showing the atom-labelling scheme, the disorder of the ethanol component, and the hydrogen bonds, drawn as dashed lines, within the asymmetric unit. Displacement ellipsoids are drawn at the 30% probability level; the major disorder component of the ethanol is drawn with full lines, and the minor component is drawn with broken lines.

**Table 1:** Hydrogen-bond geometry (Å, °) for (**I**)

*D*—H⋯*A*	*D*—H	H⋯*A*	*D*⋯*A*	*D*—H⋯*A*
N1—H1⋯N25	0.85 (2)	2.62 (2)	2.946 (2)	104.0 (16)
N21—H21⋯O51^i^	0.88 (2)	1.84 (2)	2.700 (3)	166.0 (18)
N21—H21⋯O61^i^	0.88 (2)	1.88 (3)	2.730 (12)	164 (2)
O51—H51⋯O1	0.82	1.89	2.706 (4)	175

Symmetry code: (i) 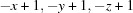.

**Table 2:** Hydrogen-bond geometry (Å, °) for (**II**)

*D*—H⋯*A*	*D*—H	H⋯*A*	*D*⋯*A*	*D*—H⋯*A*
N1—H1⋯N25	0.83 (3)	2.56 (3)	2.961 (3)	111 (2)
N21—H21⋯O51^i^	0.91 (3)	1.85 (3)	2.747 (4)	167 (2)
N21—H21⋯O61^i^	0.91 (3)	1.77 (3)	2.65 (4)	164 (3)
O51—H51⋯O1	0.82	1.94	2.754 (4)	171

Symmetry code: (i) 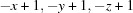.

**Table 3:** Experimental details

	(**I**)	(**II**)
Crystal data		
Chemical formula	C_19_H_15_N_7_O_3_S·C_2_H_6_O	C_20_H_17_N_7_O_3_S·C_2_H_6_O
*M*_r_	467.51	481.53
Crystal system, space group	Triclinic, *P* 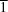	Triclinic, *P* 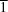
Temperature (K)	293	293
*a*, *b*, *c* (Å)	8.6313 (6), 10.8378 (9), 13.384 (1)	8.5631 (5), 11.1242 (8), 13.5632 (9)
α, β, γ (°)	66.645 (8), 79.287 (8), 85.151 (8)	70.244 (6), 76.086 (7), 84.058 (6)
*V* (Å^3^)	1129.30 (16)	1179.92 (15)
*Z*	2	2
Radiation type	Mo *K*α	Mo *K*α
μ (mm^−1^)	0.19	0.18
Crystal size (mm)	0.48 × 0.28 × 0.24	0.50 × 0.12 × 0.08

Data collection		
Diffractometer	Oxford Diffraction Xcalibur with Sapphire CCD	Oxford Diffraction Xcalibur with Sapphire CCD
Absorption correction	Multi-scan (*CrysAlis RED*; Oxford Diffraction, 2009 ▸)	Multi-scan (*CrysAlis RED*; Oxford Diffraction, 2009 ▸)
*T*_min_, *T*_max_	0.822, 0.956	0.841, 0.986
No. of measured, independent and observed [*I* > 2σ(*I*)] reflections	7829, 4464, 2985	8056, 4670, 2828
*R*_int_	0.016	0.025
(sin θ/λ)_max_ (Å^−1^)	0.618	0.618

Refinement		
*R*[*F* ^2^ > 2σ(*F* ^2^)], *wR*(*F* ^2^), *S*	0.040, 0.116, 0.96	0.062, 0.117, 1.10
No. of reflections	4464	4670
No. of parameters	317	327
No. of restraints	3	3
H-atom treatment	H atoms treated by a mixture of independent and constrained refinement	H atoms treated by a mixture of independent and constrained refinement
Δρ_max_, Δρ_min_ (e Å^−3^)	0.23, −0.22	0.19, −0.18

Computer programs: *CrysAlis CCD* and *CrysAlis RED* (Oxford Diffraction, 2009 ▸), *SHELXT* (Sheldrick, 2015*a* ▸), *SHELXL2014* (Sheldrick, 2015*b* ▸) and *PLATON* (Spek, 2020 ▸).

## supplementary crystallographic information

### N-{[4-(3-phenyl-4-sydnonylideneamino)-5-sulfanylidene-1H-1,2,4-triazol-3-yl]methyl}benzamide ethanol monosolvate (I) Crystal data

**Table:**

C_19_H_15_N_7_O_3_S·C_2_H_6_O	*Z* = 2
*M**_r_* = 467.51	*F*(000) = 488
Triclinic, *P*1	*D*_x_ = 1.375 Mg m^−^^3^
*a* = 8.6313 (6) Å	Mo *K*α radiation, λ = 0.71073 Å
*b* = 10.8378 (9) Å	Cell parameters from 4868 reflections
*c* = 13.384 (1) Å	θ = 2.9–28.0°
α = 66.645 (8)°	µ = 0.19 mm^−^^1^
β = 79.287 (8)°	*T* = 293 K
γ = 85.151 (8)°	Needle, yellow
*V* = 1129.30 (16) Å^3^	0.48 × 0.28 × 0.24 mm

### N-{[4-(3-phenyl-4-sydnonylideneamino)-5-sulfanylidene-1H-1,2,4-triazol-3-yl]methyl}benzamide ethanol monosolvate (I) Data collection

**Table:**

Oxford Diffraction Xcalibur with Sapphire CCD diffractometer	4464 independent reflections
Radiation source: Enhance (Mo) X-ray Source	2985 reflections with *I* > 2σ(*I*)
Graphite monochromator	*R*_int_ = 0.016
ω scans	θ_max_ = 26.1°, θ_min_ = 2.9°
Absorption correction: multi-scan (CrysAlis RED; Oxford Diffraction, 2009)	*h* = −10→6
*T*_min_ = 0.822, *T*_max_ = 0.956	*k* = −13→12
7829 measured reflections	*l* = −16→16

### N-{[4-(3-phenyl-4-sydnonylideneamino)-5-sulfanylidene-1H-1,2,4-triazol-3-yl]methyl}benzamide ethanol monosolvate (I) Refinement

**Table:**

Refinement on *F*^2^	Primary atom site location: difference Fourier map
Least-squares matrix: full	Hydrogen site location: mixed
*R*[*F*^2^ > 2σ(*F*^2^)] = 0.040	H atoms treated by a mixture of independent and constrained refinement
*wR*(*F*^2^) = 0.116	*w* = 1/[σ^2^(*F*_o_^2^) + (0.0712*P*)^2^] where *P* = (*F*_o_^2^ + 2*F*_c_^2^)/3
*S* = 0.96	(Δ/σ)_max_ < 0.001
4464 reflections	Δρ_max_ = 0.23 e Å^−^^3^
317 parameters	Δρ_min_ = −0.22 e Å^−^^3^
3 restraints	

### N-{[4-(3-phenyl-4-sydnonylideneamino)-5-sulfanylidene-1H-1,2,4-triazol-3-yl]methyl}benzamide ethanol monosolvate (I) Special details

**Table:**

Geometry. All esds (except the esd in the dihedral angle between two l.s. planes) are estimated using the full covariance matrix. The cell esds are taken into account individually in the estimation of esds in distances, angles and torsion angles; correlations between esds in cell parameters are only used when they are defined by crystal symmetry. An approximate (isotropic) treatment of cell esds is used for estimating esds involving l.s. planes.

### N-{[4-(3-phenyl-4-sydnonylideneamino)-5-sulfanylidene-1H-1,2,4-triazol-3-yl]methyl}benzamide ethanol monosolvate (I) Fractional atomic coordinates and isotropic or equivalent isotropic displacement parameters (Å^2^)

**Table:**

	*x*	*y*	*z*	*U*_iso_*/*U*_eq_	Occ. (<1)
C1	0.4087 (2)	0.49774 (19)	0.18161 (16)	0.0539 (5)	
O1	0.4564 (2)	0.57411 (15)	0.21616 (15)	0.0905 (5)	
N1	0.44135 (19)	0.36690 (16)	0.22477 (13)	0.0521 (4)	
H1	0.400 (2)	0.316 (2)	0.2026 (17)	0.063*	
C11	0.3163 (2)	0.54838 (18)	0.08944 (15)	0.0511 (5)	
C12	0.2787 (2)	0.4703 (2)	0.03721 (17)	0.0590 (5)	
H12	0.3096	0.3804	0.0605	0.071*	
C13	0.1954 (3)	0.5253 (3)	−0.04951 (19)	0.0728 (6)	
H13	0.1705	0.4718	−0.0840	0.087*	
C14	0.1495 (3)	0.6565 (3)	−0.0849 (2)	0.0770 (7)	
H14	0.0932	0.6927	−0.1432	0.092*	
C15	0.1865 (3)	0.7346 (3)	−0.0343 (2)	0.0879 (8)	
H15	0.1558	0.8247	−0.0586	0.106*	
C16	0.2686 (3)	0.6813 (2)	0.05221 (19)	0.0766 (7)	
H16	0.2924	0.7356	0.0862	0.092*	
C17	0.5456 (2)	0.31146 (19)	0.30517 (16)	0.0548 (5)	
H17A	0.6128	0.3825	0.2987	0.066*	
H17B	0.6131	0.2441	0.2875	0.066*	
N21	0.39481 (18)	0.18933 (16)	0.59238 (14)	0.0514 (4)	
H21	0.397 (2)	0.1937 (19)	0.6562 (18)	0.062*	
N22	0.48632 (18)	0.27926 (16)	0.50167 (14)	0.0546 (4)	
C23	0.46533 (19)	0.24940 (16)	0.42114 (15)	0.0437 (4)	
N24	0.36560 (15)	0.14207 (13)	0.45836 (11)	0.0389 (3)	
C25	0.31748 (19)	0.10342 (16)	0.57162 (14)	0.0413 (4)	
S25	0.18954 (6)	−0.01347 (5)	0.65906 (4)	0.05549 (17)	
N25	0.32368 (16)	0.10047 (14)	0.38197 (11)	0.0433 (3)	
C26	0.26974 (18)	−0.01806 (16)	0.41643 (14)	0.0395 (4)	
H26	0.2588	−0.0751	0.4906	0.047*	
O31	0.1874 (2)	−0.08129 (15)	0.18574 (12)	0.0762 (5)	
N32	0.1439 (2)	−0.19906 (17)	0.27290 (15)	0.0663 (5)	
N33	0.17026 (16)	−0.18125 (14)	0.35876 (12)	0.0455 (4)	
C34	0.2272 (2)	−0.05913 (16)	0.33674 (15)	0.0429 (4)	
C35	0.2393 (3)	0.0120 (2)	0.22153 (17)	0.0610 (5)	
O35	0.2818 (2)	0.12192 (15)	0.15706 (12)	0.0876 (5)	
C41	0.1316 (2)	−0.28902 (17)	0.46537 (15)	0.0448 (4)	
C42	0.1955 (2)	−0.41464 (19)	0.4829 (2)	0.0605 (5)	
H42	0.2644	−0.4315	0.4274	0.073*	
C43	0.1543 (3)	−0.5148 (2)	0.5855 (2)	0.0753 (7)	
H43	0.1959	−0.6009	0.5993	0.090*	
C44	0.0533 (3)	−0.4903 (2)	0.6675 (2)	0.0752 (7)	
H44	0.0261	−0.5595	0.7362	0.090*	
C45	−0.0075 (2)	−0.3635 (2)	0.64828 (18)	0.0650 (6)	
H45	−0.0746	−0.3464	0.7044	0.078*	
C46	0.0303 (2)	−0.26166 (18)	0.54647 (16)	0.0511 (5)	
H46	−0.0118	−0.1757	0.5326	0.061*	
O51	0.5469 (4)	0.8063 (3)	0.21769 (15)	0.0720 (8)	0.836 (6)
H51	0.5236	0.7365	0.2142	0.108*	0.836 (6)
C51	0.6351 (5)	0.8888 (4)	0.1149 (3)	0.0896 (11)	0.836 (6)
H51A	0.6227	0.9819	0.1068	0.107*	0.836 (6)
H51B	0.5930	0.8805	0.0556	0.107*	0.836 (6)
C52	0.7995 (5)	0.8544 (7)	0.1049 (4)	0.1074 (13)	0.836 (6)
H52A	0.8533	0.9122	0.0340	0.161*	0.836 (6)
H52B	0.8126	0.7626	0.1119	0.161*	0.836 (6)
H52C	0.8427	0.8652	0.1620	0.161*	0.836 (6)
O61	0.6330 (19)	0.7535 (13)	0.2239 (8)	0.0720 (8)	0.164 (6)
H61	0.5772	0.6975	0.2215	0.108*	0.164 (6)
C61	0.672 (3)	0.834 (2)	0.1102 (12)	0.0896 (11)	0.164 (6)
H61A	0.6170	0.9198	0.0952	0.107*	0.164 (6)
H61B	0.6343	0.7906	0.0684	0.107*	0.164 (6)
C62	0.837 (3)	0.858 (4)	0.073 (2)	0.1074 (13)	0.164 (6)
H62A	0.8914	0.8253	0.1348	0.161*	0.164 (6)
H62B	0.8544	0.9526	0.0330	0.161*	0.164 (6)
H62C	0.8765	0.8117	0.0246	0.161*	0.164 (6)

### N-{[4-(3-phenyl-4-sydnonylideneamino)-5-sulfanylidene-1H-1,2,4-triazol-3-yl]methyl}benzamide ethanol monosolvate (I) Atomic displacement parameters (Å^2^)

**Table:**

	*U*^11^	*U*^22^	*U*^33^	*U*^12^	*U*^13^	*U*^23^
C1	0.0647 (12)	0.0410 (11)	0.0513 (11)	−0.0048 (9)	0.0002 (10)	−0.0164 (9)
O1	0.1374 (15)	0.0516 (9)	0.0993 (13)	0.0019 (9)	−0.0475 (11)	−0.0355 (9)
N1	0.0604 (10)	0.0401 (9)	0.0516 (10)	−0.0061 (7)	−0.0088 (8)	−0.0124 (7)
C11	0.0570 (11)	0.0408 (10)	0.0444 (10)	−0.0021 (8)	0.0048 (9)	−0.0105 (8)
C12	0.0591 (12)	0.0497 (12)	0.0605 (13)	−0.0074 (9)	−0.0033 (10)	−0.0147 (10)
C13	0.0716 (15)	0.0766 (17)	0.0638 (14)	−0.0180 (12)	−0.0094 (12)	−0.0181 (13)
C14	0.0673 (14)	0.0774 (17)	0.0603 (14)	−0.0062 (12)	−0.0078 (11)	0.0002 (13)
C15	0.105 (2)	0.0605 (15)	0.0736 (17)	0.0198 (14)	−0.0149 (15)	−0.0046 (13)
C16	0.1100 (19)	0.0488 (13)	0.0644 (15)	0.0088 (12)	−0.0134 (14)	−0.0176 (11)
C17	0.0500 (11)	0.0454 (11)	0.0629 (13)	−0.0073 (9)	−0.0064 (10)	−0.0146 (10)
N21	0.0605 (10)	0.0528 (10)	0.0502 (10)	0.0000 (8)	−0.0180 (8)	−0.0259 (8)
N22	0.0569 (10)	0.0506 (10)	0.0643 (11)	−0.0038 (8)	−0.0183 (8)	−0.0266 (9)
C23	0.0407 (9)	0.0368 (9)	0.0544 (11)	−0.0010 (7)	−0.0118 (8)	−0.0171 (8)
N24	0.0435 (8)	0.0347 (7)	0.0412 (8)	−0.0010 (6)	−0.0108 (6)	−0.0159 (6)
C25	0.0457 (10)	0.0378 (9)	0.0437 (10)	0.0100 (7)	−0.0145 (8)	−0.0184 (8)
S25	0.0654 (3)	0.0496 (3)	0.0435 (3)	−0.0021 (2)	−0.0044 (2)	−0.0115 (2)
N25	0.0521 (8)	0.0399 (8)	0.0405 (8)	−0.0051 (6)	−0.0073 (7)	−0.0176 (7)
C26	0.0426 (9)	0.0340 (9)	0.0411 (9)	0.0017 (7)	−0.0084 (7)	−0.0135 (7)
O31	0.1185 (13)	0.0688 (10)	0.0512 (9)	−0.0207 (9)	−0.0151 (8)	−0.0293 (8)
N32	0.0916 (13)	0.0572 (11)	0.0617 (11)	−0.0142 (9)	−0.0135 (10)	−0.0322 (9)
N33	0.0507 (8)	0.0413 (8)	0.0513 (9)	−0.0033 (7)	−0.0087 (7)	−0.0246 (7)
C34	0.0499 (10)	0.0361 (9)	0.0449 (10)	−0.0041 (8)	−0.0060 (8)	−0.0180 (8)
C35	0.0871 (15)	0.0525 (13)	0.0485 (12)	−0.0108 (11)	−0.0104 (11)	−0.0234 (10)
O35	0.1473 (16)	0.0598 (10)	0.0485 (9)	−0.0296 (10)	−0.0126 (9)	−0.0097 (8)
C41	0.0442 (10)	0.0352 (9)	0.0560 (11)	−0.0055 (7)	−0.0127 (8)	−0.0161 (8)
C42	0.0574 (12)	0.0427 (11)	0.0857 (16)	0.0009 (9)	−0.0145 (11)	−0.0286 (11)
C43	0.0738 (15)	0.0357 (12)	0.109 (2)	0.0044 (10)	−0.0313 (14)	−0.0139 (13)
C44	0.0765 (15)	0.0523 (14)	0.0777 (16)	−0.0121 (11)	−0.0255 (13)	0.0023 (12)
C45	0.0615 (13)	0.0629 (14)	0.0607 (13)	−0.0105 (10)	−0.0060 (10)	−0.0137 (11)
C46	0.0474 (10)	0.0428 (11)	0.0598 (12)	0.0009 (8)	−0.0105 (9)	−0.0161 (9)
O51	0.095 (2)	0.0762 (16)	0.0573 (10)	−0.0211 (14)	−0.0090 (11)	−0.0365 (10)
C51	0.101 (3)	0.096 (3)	0.0654 (18)	0.001 (2)	−0.0205 (17)	−0.0220 (19)
C52	0.104 (4)	0.140 (3)	0.071 (3)	0.000 (3)	−0.005 (2)	−0.038 (3)
O61	0.095 (2)	0.0762 (16)	0.0573 (10)	−0.0211 (14)	−0.0090 (11)	−0.0365 (10)
C61	0.101 (3)	0.096 (3)	0.0654 (18)	0.001 (2)	−0.0205 (17)	−0.0220 (19)
C62	0.104 (4)	0.140 (3)	0.071 (3)	0.000 (3)	−0.005 (2)	−0.038 (3)

### N-{[4-(3-phenyl-4-sydnonylideneamino)-5-sulfanylidene-1H-1,2,4-triazol-3-yl]methyl}benzamide ethanol monosolvate (I) Geometric parameters (Å, º)

**Table:**

C1—O1	1.225 (2)	N32—N33	1.300 (2)
C1—N1	1.329 (2)	N33—C34	1.352 (2)
C1—C11	1.488 (3)	N33—C41	1.442 (2)
N1—C17	1.444 (2)	C34—C35	1.412 (3)
N1—H1	0.851 (19)	C35—O35	1.200 (2)
C11—C16	1.378 (3)	C41—C42	1.371 (2)
C11—C12	1.382 (3)	C41—C46	1.374 (3)
C12—C13	1.382 (3)	C42—C43	1.375 (3)
C12—H12	0.9300	C42—H42	0.9300
C13—C14	1.358 (3)	C43—C44	1.369 (3)
C13—H13	0.9300	C43—H43	0.9300
C14—C15	1.364 (3)	C44—C45	1.370 (3)
C14—H14	0.9300	C44—H44	0.9300
C15—C16	1.372 (3)	C45—C46	1.373 (3)
C15—H15	0.9300	C45—H45	0.9300
C16—H16	0.9300	C46—H46	0.9300
C17—C23	1.481 (3)	O51—C51	1.422 (3)
C17—H17A	0.9700	O51—H51	0.8200
C17—H17B	0.9700	C51—C52	1.432 (5)
N21—C25	1.330 (2)	C51—H51A	0.9700
N21—N22	1.372 (2)	C51—H51B	0.9700
N21—H21	0.88 (2)	C52—H52A	0.9600
N22—C23	1.288 (2)	C52—H52B	0.9600
C23—N24	1.376 (2)	C52—H52C	0.9600
N24—N25	1.3821 (18)	O61—C61	1.413 (9)
N24—C25	1.392 (2)	O61—H61	0.8198
C25—S25	1.6657 (18)	C61—C62	1.431 (10)
N25—C26	1.277 (2)	C61—H61A	0.9700
C26—C34	1.421 (2)	C61—H61B	0.9700
C26—H26	0.9300	C62—H62A	0.9600
O31—N32	1.367 (2)	C62—H62B	0.9600
O31—C35	1.412 (2)	C62—H62C	0.9600
			
O1—C1—N1	120.3 (2)	C34—N33—C41	127.36 (15)
O1—C1—C11	121.36 (18)	N33—C34—C35	105.95 (15)
N1—C1—C11	118.31 (17)	N33—C34—C26	124.92 (16)
C1—N1—C17	121.94 (16)	C35—C34—C26	129.09 (16)
C1—N1—H1	118.0 (14)	O35—C35—O31	121.02 (18)
C17—N1—H1	120.0 (14)	O35—C35—C34	135.49 (18)
C16—C11—C12	118.0 (2)	O31—C35—C34	103.48 (16)
C16—C11—C1	118.33 (18)	C42—C41—C46	122.09 (18)
C12—C11—C1	123.63 (18)	C42—C41—N33	119.66 (17)
C13—C12—C11	120.3 (2)	C46—C41—N33	118.25 (15)
C13—C12—H12	119.8	C41—C42—C43	117.8 (2)
C11—C12—H12	119.8	C41—C42—H42	121.1
C14—C13—C12	120.7 (2)	C43—C42—H42	121.1
C14—C13—H13	119.6	C44—C43—C42	121.3 (2)
C12—C13—H13	119.6	C44—C43—H43	119.4
C13—C14—C15	119.5 (2)	C42—C43—H43	119.4
C13—C14—H14	120.2	C43—C44—C45	119.8 (2)
C15—C14—H14	120.2	C43—C44—H44	120.1
C14—C15—C16	120.4 (2)	C45—C44—H44	120.1
C14—C15—H15	119.8	C44—C45—C46	120.3 (2)
C16—C15—H15	119.8	C44—C45—H45	119.9
C15—C16—C11	121.0 (2)	C46—C45—H45	119.9
C15—C16—H16	119.5	C45—C46—C41	118.77 (18)
C11—C16—H16	119.5	C45—C46—H46	120.6
N1—C17—C23	114.88 (15)	C41—C46—H46	120.6
N1—C17—H17A	108.5	C51—O51—H51	109.5
C23—C17—H17A	108.5	O51—C51—C52	112.8 (3)
N1—C17—H17B	108.5	O51—C51—H51A	109.0
C23—C17—H17B	108.5	C52—C51—H51A	109.0
H17A—C17—H17B	107.5	O51—C51—H51B	109.0
C25—N21—N22	114.74 (15)	C52—C51—H51B	109.0
C25—N21—H21	128.3 (13)	H51A—C51—H51B	107.8
N22—N21—H21	116.9 (13)	C51—C52—H52A	109.5
C23—N22—N21	104.27 (14)	C51—C52—H52B	109.5
N22—C23—N24	110.68 (16)	H52A—C52—H52B	109.5
N22—C23—C17	126.19 (15)	C51—C52—H52C	109.5
N24—C23—C17	122.94 (16)	H52A—C52—H52C	109.5
C23—N24—N25	118.51 (14)	H52B—C52—H52C	109.5
C23—N24—C25	108.53 (14)	C61—O61—H61	100.2
N25—N24—C25	132.76 (14)	O61—C61—C62	113.9 (14)
N21—C25—N24	101.77 (15)	O61—C61—H61A	108.8
N21—C25—S25	128.17 (14)	C62—C61—H61A	108.8
N24—C25—S25	129.98 (13)	O61—C61—H61B	108.8
C26—N25—N24	118.01 (14)	C62—C61—H61B	108.8
N25—C26—C34	117.24 (16)	H61A—C61—H61B	107.7
N25—C26—H26	121.4	C61—C62—H62A	109.5
C34—C26—H26	121.4	C61—C62—H62B	109.5
N32—O31—C35	111.00 (14)	H62A—C62—H62B	109.5
N33—N32—O31	104.83 (13)	C61—C62—H62C	109.5
N32—N33—C34	114.72 (16)	H62A—C62—H62C	109.5
N32—N33—C41	117.88 (14)	H62B—C62—H62C	109.5
			
O1—C1—N1—C17	−6.2 (3)	C23—N24—N25—C26	160.75 (15)
C11—C1—N1—C17	172.55 (16)	C25—N24—N25—C26	−25.1 (3)
O1—C1—C11—C16	−7.8 (3)	N24—N25—C26—C34	179.98 (14)
N1—C1—C11—C16	173.48 (18)	C35—O31—N32—N33	−1.1 (2)
O1—C1—C11—C12	170.63 (19)	O31—N32—N33—C34	0.7 (2)
N1—C1—C11—C12	−8.1 (3)	O31—N32—N33—C41	178.55 (15)
C16—C11—C12—C13	−0.1 (3)	N32—N33—C34—C35	0.1 (2)
C1—C11—C12—C13	−178.57 (18)	C41—N33—C34—C35	−177.57 (17)
C11—C12—C13—C14	0.1 (3)	N32—N33—C34—C26	−177.83 (16)
C12—C13—C14—C15	0.1 (3)	C41—N33—C34—C26	4.5 (3)
C13—C14—C15—C16	−0.4 (4)	N25—C26—C34—N33	179.15 (16)
C14—C15—C16—C11	0.4 (4)	N25—C26—C34—C35	1.7 (3)
C12—C11—C16—C15	−0.1 (3)	N32—O31—C35—O35	−179.6 (2)
C1—C11—C16—C15	178.4 (2)	N32—O31—C35—C34	1.2 (2)
C1—N1—C17—C23	100.0 (2)	N33—C34—C35—O35	−179.8 (3)
C25—N21—N22—C23	0.3 (2)	C26—C34—C35—O35	−2.0 (4)
N21—N22—C23—N24	−1.01 (19)	N33—C34—C35—O31	−0.7 (2)
N21—N22—C23—C17	−176.04 (16)	C26—C34—C35—O31	177.04 (17)
N1—C17—C23—N22	−124.95 (19)	N32—N33—C41—C42	55.7 (2)
N1—C17—C23—N24	60.6 (2)	C34—N33—C41—C42	−126.68 (19)
N22—C23—N24—N25	176.86 (14)	N32—N33—C41—C46	−124.04 (18)
C17—C23—N24—N25	−7.9 (2)	C34—N33—C41—C46	53.5 (2)
N22—C23—N24—C25	1.4 (2)	C46—C41—C42—C43	0.4 (3)
C17—C23—N24—C25	176.60 (16)	N33—C41—C42—C43	−179.38 (17)
N22—N21—C25—N24	0.50 (19)	C41—C42—C43—C44	−0.2 (3)
N22—N21—C25—S25	−176.58 (13)	C42—C43—C44—C45	−0.6 (3)
C23—N24—C25—N21	−1.08 (17)	C43—C44—C45—C46	1.2 (3)
N25—N24—C25—N21	−175.67 (16)	C44—C45—C46—C41	−0.9 (3)
C23—N24—C25—S25	175.93 (13)	C42—C41—C46—C45	0.2 (3)
N25—N24—C25—S25	1.3 (3)	N33—C41—C46—C45	179.94 (15)

### N-{[4-(3-phenyl-4-sydnonylideneamino)-5-sulfanylidene-1H-1,2,4-triazol-3-yl]methyl}benzamide ethanol monosolvate (I) Hydrogen-bond geometry (Å, º)

**Table:**

*D*—H···*A*	*D*—H	H···*A*	*D*···*A*	*D*—H···*A*
N1—H1···N25	0.85 (2)	2.62 (2)	2.946 (2)	104.0 (16)
N21—H21···O51^i^	0.88 (2)	1.84 (2)	2.700 (3)	166.0 (18)
N21—H21···O61^i^	0.88 (2)	1.88 (3)	2.730 (12)	164 (2)
O51—H51···O1	0.82	1.89	2.706 (4)	175
O61—H61···O1	0.82	1.79	2.614 (16)	180
C12—H12···O35	0.93	2.59	3.468 (3)	158

Symmetry code: (i) −*x*+1, −*y*+1, −*z*+1.

### N-({4-[3-(4-Methylphenyl)-4-sydnonylideneamino]-5-sulfanylidene-1H-1,2,4-triazol-3-yl}methyl)benzamide ethanol monosolvate (II) Crystal data

**Table:**

C_20_H_17_N_7_O_3_S·C_2_H_6_O	*Z* = 2
*M**_r_* = 481.53	*F*(000) = 504
Triclinic, *P*1	*D*_x_ = 1.355 Mg m^−^^3^
*a* = 8.5631 (5) Å	Mo *K*α radiation, λ = 0.71073 Å
*b* = 11.1242 (8) Å	Cell parameters from 5107 reflections
*c* = 13.5632 (9) Å	θ = 2.6–28.0°
α = 70.244 (6)°	µ = 0.18 mm^−^^1^
β = 76.086 (7)°	*T* = 293 K
γ = 84.058 (6)°	Needle, yellow
*V* = 1179.92 (15) Å^3^	0.50 × 0.12 × 0.08 mm

### N-({4-[3-(4-Methylphenyl)-4-sydnonylideneamino]-5-sulfanylidene-1H-1,2,4-triazol-3-yl}methyl)benzamide ethanol monosolvate (II) Data collection

**Table:**

Oxford Diffraction Xcalibur with Sapphire CCD diffractometer	4670 independent reflections
Radiation source: Enhance (Mo) X-ray Source	2828 reflections with *I* > 2σ(*I*)
Graphite monochromator	*R*_int_ = 0.025
ω scans	θ_max_ = 26.1°, θ_min_ = 2.6°
Absorption correction: multi-scan (CrysAlis RED; Oxford Diffraction, 2009)	*h* = −10→5
*T*_min_ = 0.841, *T*_max_ = 0.986	*k* = −13→13
8056 measured reflections	*l* = −16→15

### N-({4-[3-(4-Methylphenyl)-4-sydnonylideneamino]-5-sulfanylidene-1H-1,2,4-triazol-3-yl}methyl)benzamide ethanol monosolvate (II) Refinement

**Table:**

Refinement on *F*^2^	Primary atom site location: difference Fourier map
Least-squares matrix: full	Hydrogen site location: mixed
*R*[*F*^2^ > 2σ(*F*^2^)] = 0.062	H atoms treated by a mixture of independent and constrained refinement
*wR*(*F*^2^) = 0.117	*w* = 1/[σ^2^(*F*_o_^2^) + (0.0283*P*)^2^ + 0.4689*P*] where *P* = (*F*_o_^2^ + 2*F*_c_^2^)/3
*S* = 1.10	(Δ/σ)_max_ < 0.001
4670 reflections	Δρ_max_ = 0.19 e Å^−^^3^
327 parameters	Δρ_min_ = −0.18 e Å^−^^3^
3 restraints	

### N-({4-[3-(4-Methylphenyl)-4-sydnonylideneamino]-5-sulfanylidene-1H-1,2,4-triazol-3-yl}methyl)benzamide ethanol monosolvate (II) Special details

**Table:**

Geometry. All esds (except the esd in the dihedral angle between two l.s. planes) are estimated using the full covariance matrix. The cell esds are taken into account individually in the estimation of esds in distances, angles and torsion angles; correlations between esds in cell parameters are only used when they are defined by crystal symmetry. An approximate (isotropic) treatment of cell esds is used for estimating esds involving l.s. planes.

### N-({4-[3-(4-Methylphenyl)-4-sydnonylideneamino]-5-sulfanylidene-1H-1,2,4-triazol-3-yl}methyl)benzamide ethanol monosolvate (II) Fractional atomic coordinates and isotropic or equivalent isotropic displacement parameters (Å^2^)

**Table:**

	*x*	*y*	*z*	*U*_iso_*/*U*_eq_	Occ. (<1)
C1	0.4320 (3)	0.4989 (3)	0.1887 (2)	0.0516 (7)	
O1	0.5007 (3)	0.57130 (19)	0.21463 (17)	0.0728 (6)	
N1	0.4417 (3)	0.3724 (2)	0.2369 (2)	0.0571 (7)	
H1	0.390 (3)	0.325 (3)	0.221 (2)	0.069*	
C11	0.3385 (3)	0.5462 (3)	0.1039 (2)	0.0498 (7)	
C12	0.2858 (3)	0.4682 (3)	0.0582 (2)	0.0593 (8)	
H12	0.3071	0.3807	0.0818	0.071*	
C13	0.2022 (4)	0.5194 (4)	−0.0219 (3)	0.0728 (10)	
H13	0.1675	0.4663	−0.0521	0.087*	
C14	0.1696 (4)	0.6474 (4)	−0.0573 (3)	0.0840 (11)	
H14	0.1124	0.6816	−0.1112	0.101*	
C15	0.2216 (5)	0.7253 (4)	−0.0129 (3)	0.0894 (12)	
H15	0.1991	0.8127	−0.0366	0.107*	
C16	0.3065 (4)	0.6754 (3)	0.0662 (3)	0.0722 (9)	
H16	0.3430	0.7295	0.0947	0.087*	
C17	0.5493 (3)	0.3149 (3)	0.3086 (2)	0.0596 (8)	
H17A	0.6172	0.3808	0.3063	0.072*	
H17B	0.6187	0.2526	0.2822	0.072*	
N21	0.4015 (3)	0.1866 (2)	0.5901 (2)	0.0560 (7)	
H21	0.401 (3)	0.184 (3)	0.658 (2)	0.067*	
N22	0.4901 (3)	0.2760 (2)	0.5037 (2)	0.0597 (7)	
C23	0.4687 (3)	0.2512 (3)	0.4215 (2)	0.0492 (7)	
N24	0.3715 (2)	0.14807 (19)	0.45373 (18)	0.0430 (5)	
C25	0.3256 (3)	0.1057 (3)	0.5646 (2)	0.0465 (7)	
S25	0.20592 (10)	−0.01225 (8)	0.64723 (6)	0.0607 (2)	
N25	0.3260 (3)	0.1148 (2)	0.37505 (17)	0.0463 (6)	
C26	0.2769 (3)	0.0009 (2)	0.4004 (2)	0.0427 (6)	
H26	0.2733	−0.0578	0.4686	0.051*	
O31	0.1843 (3)	−0.0446 (2)	0.16972 (16)	0.0798 (7)	
N32	0.1452 (3)	−0.1594 (2)	0.2474 (2)	0.0666 (7)	
N33	0.1738 (3)	−0.1471 (2)	0.33327 (18)	0.0463 (6)	
C34	0.2283 (3)	−0.0315 (2)	0.3201 (2)	0.0449 (7)	
C35	0.2365 (4)	0.0409 (3)	0.2114 (3)	0.0667 (9)	
O35	0.2748 (3)	0.1471 (2)	0.15540 (18)	0.0986 (9)	
C41	0.1448 (3)	−0.2546 (2)	0.4303 (2)	0.0421 (6)	
C42	0.2134 (3)	−0.3709 (3)	0.4292 (2)	0.0541 (8)	
H42	0.2745	−0.3818	0.3660	0.065*	
C43	0.1898 (4)	−0.4716 (3)	0.5243 (3)	0.0607 (8)	
H43	0.2357	−0.5511	0.5244	0.073*	
C44	0.1002 (3)	−0.4577 (3)	0.6190 (3)	0.0547 (8)	
C45	0.0311 (3)	−0.3401 (3)	0.6162 (2)	0.0579 (8)	
H45	−0.0315	−0.3291	0.6790	0.069*	
C46	0.0520 (3)	−0.2377 (3)	0.5224 (2)	0.0505 (7)	
H46	0.0041	−0.1586	0.5216	0.061*	
C47	0.0841 (4)	−0.5680 (3)	0.7222 (3)	0.0829 (11)	
H47A	0.0051	−0.5465	0.7775	0.124*	
H47B	0.1859	−0.5857	0.7429	0.124*	
H47C	0.0512	−0.6421	0.7121	0.124*	
O51	0.5508 (4)	0.8106 (3)	0.2175 (2)	0.0639 (10)	0.906 (6)
H51	0.5341	0.7434	0.2098	0.096*	0.906 (6)
C51	0.6322 (6)	0.8952 (5)	0.1173 (3)	0.0883 (16)	0.906 (6)
H51A	0.6089	0.9824	0.1174	0.106*	0.906 (6)
H51B	0.5898	0.8854	0.0604	0.106*	0.906 (6)
C52	0.8062 (5)	0.8748 (5)	0.0935 (3)	0.0981 (17)	0.906 (6)
H52A	0.8537	0.9409	0.0295	0.147*	0.906 (6)
H52B	0.8311	0.7932	0.0831	0.147*	0.906 (6)
H52C	0.8486	0.8768	0.1524	0.147*	0.906 (6)
O61	0.599 (5)	0.773 (3)	0.228 (3)	0.0639 (10)	0.094 (6)
H61	0.5670	0.7079	0.2237	0.096*	0.094 (6)
C61	0.663 (8)	0.833 (3)	0.116 (3)	0.0883 (16)	0.094 (6)
H61A	0.5833	0.8341	0.0758	0.106*	0.094 (6)
H61B	0.7564	0.7835	0.0923	0.106*	0.094 (6)
C62	0.711 (6)	0.963 (3)	0.095 (3)	0.0981 (17)	0.094 (6)
H62A	0.8222	0.9729	0.0598	0.147*	0.094 (6)
H62B	0.6937	0.9801	0.1619	0.147*	0.094 (6)
H62C	0.6465	1.0224	0.0501	0.147*	0.094 (6)

### N-({4-[3-(4-Methylphenyl)-4-sydnonylideneamino]-5-sulfanylidene-1H-1,2,4-triazol-3-yl}methyl)benzamide ethanol monosolvate (II) Atomic displacement parameters (Å^2^)

**Table:**

	*U*^11^	*U*^22^	*U*^33^	*U*^12^	*U*^13^	*U*^23^
C1	0.0548 (18)	0.0450 (18)	0.0514 (18)	−0.0072 (15)	0.0021 (15)	−0.0186 (15)
O1	0.1006 (17)	0.0496 (13)	0.0801 (16)	−0.0097 (12)	−0.0273 (13)	−0.0287 (12)
N1	0.0597 (17)	0.0467 (16)	0.0666 (18)	−0.0120 (13)	−0.0199 (13)	−0.0131 (13)
C11	0.0483 (17)	0.0459 (17)	0.0498 (18)	−0.0020 (14)	0.0020 (14)	−0.0172 (14)
C12	0.0555 (18)	0.0549 (19)	0.064 (2)	−0.0025 (15)	−0.0071 (16)	−0.0188 (17)
C13	0.066 (2)	0.084 (3)	0.069 (2)	−0.010 (2)	−0.0138 (19)	−0.025 (2)
C14	0.068 (2)	0.097 (3)	0.068 (2)	−0.004 (2)	−0.0143 (19)	−0.002 (2)
C15	0.092 (3)	0.061 (2)	0.098 (3)	0.007 (2)	−0.024 (2)	−0.004 (2)
C16	0.084 (2)	0.051 (2)	0.078 (2)	0.0000 (18)	−0.016 (2)	−0.0178 (18)
C17	0.0545 (18)	0.0515 (18)	0.072 (2)	−0.0104 (15)	−0.0173 (17)	−0.0146 (17)
N21	0.0683 (17)	0.0571 (16)	0.0571 (17)	0.0055 (13)	−0.0268 (15)	−0.0301 (15)
N22	0.0655 (16)	0.0506 (15)	0.0738 (19)	−0.0021 (13)	−0.0280 (15)	−0.0251 (14)
C23	0.0477 (17)	0.0448 (17)	0.064 (2)	0.0002 (14)	−0.0205 (15)	−0.0236 (15)
N24	0.0470 (13)	0.0401 (13)	0.0495 (15)	−0.0034 (11)	−0.0153 (11)	−0.0206 (11)
C25	0.0500 (17)	0.0463 (17)	0.0499 (18)	0.0101 (14)	−0.0188 (14)	−0.0218 (14)
S25	0.0708 (5)	0.0570 (5)	0.0516 (5)	−0.0006 (4)	−0.0106 (4)	−0.0165 (4)
N25	0.0535 (14)	0.0433 (14)	0.0495 (14)	−0.0051 (11)	−0.0146 (11)	−0.0210 (11)
C26	0.0470 (16)	0.0383 (16)	0.0436 (16)	0.0013 (13)	−0.0103 (13)	−0.0148 (13)
O31	0.139 (2)	0.0618 (14)	0.0462 (13)	−0.0308 (14)	−0.0296 (13)	−0.0119 (11)
N32	0.103 (2)	0.0557 (16)	0.0506 (16)	−0.0197 (15)	−0.0224 (15)	−0.0203 (13)
N33	0.0560 (14)	0.0412 (13)	0.0461 (14)	−0.0067 (11)	−0.0113 (11)	−0.0180 (11)
C34	0.0567 (17)	0.0366 (15)	0.0446 (17)	−0.0055 (13)	−0.0125 (14)	−0.0150 (13)
C35	0.099 (3)	0.055 (2)	0.055 (2)	−0.0181 (19)	−0.0223 (18)	−0.0199 (17)
O35	0.180 (3)	0.0552 (15)	0.0578 (15)	−0.0416 (16)	−0.0319 (16)	−0.0002 (12)
C41	0.0437 (16)	0.0387 (15)	0.0476 (17)	−0.0048 (13)	−0.0115 (13)	−0.0168 (13)
C42	0.0572 (18)	0.0455 (17)	0.065 (2)	−0.0037 (15)	−0.0080 (15)	−0.0273 (16)
C43	0.062 (2)	0.0318 (16)	0.088 (3)	0.0025 (14)	−0.0227 (18)	−0.0164 (17)
C44	0.0501 (18)	0.0480 (18)	0.063 (2)	−0.0117 (15)	−0.0184 (16)	−0.0075 (16)
C45	0.0551 (18)	0.060 (2)	0.0523 (19)	−0.0048 (16)	−0.0047 (15)	−0.0136 (16)
C46	0.0506 (17)	0.0416 (16)	0.058 (2)	0.0044 (14)	−0.0095 (15)	−0.0184 (15)
C47	0.086 (3)	0.062 (2)	0.084 (3)	−0.0187 (19)	−0.026 (2)	0.0074 (19)
O51	0.089 (2)	0.056 (2)	0.0543 (15)	−0.0169 (16)	−0.0188 (14)	−0.0212 (14)
C51	0.103 (4)	0.070 (4)	0.079 (3)	−0.021 (3)	−0.032 (3)	0.005 (3)
C52	0.095 (4)	0.100 (4)	0.070 (3)	−0.004 (3)	−0.015 (2)	0.007 (3)
O61	0.089 (2)	0.056 (2)	0.0543 (15)	−0.0169 (16)	−0.0188 (14)	−0.0212 (14)
C61	0.103 (4)	0.070 (4)	0.079 (3)	−0.021 (3)	−0.032 (3)	0.005 (3)
C62	0.095 (4)	0.100 (4)	0.070 (3)	−0.004 (3)	−0.015 (2)	0.007 (3)

### N-({4-[3-(4-Methylphenyl)-4-sydnonylideneamino]-5-sulfanylidene-1H-1,2,4-triazol-3-yl}methyl)benzamide ethanol monosolvate (II) Geometric parameters (Å, º)

**Table:**

C1—O1	1.228 (3)	N33—C41	1.436 (3)
C1—N1	1.340 (3)	C34—C35	1.409 (4)
C1—C11	1.479 (4)	C35—O35	1.198 (3)
N1—C17	1.446 (4)	C41—C42	1.368 (3)
N1—H1	0.83 (3)	C41—C46	1.370 (4)
C11—C16	1.375 (4)	C42—C43	1.379 (4)
C11—C12	1.386 (4)	C42—H42	0.9300
C12—C13	1.373 (4)	C43—C44	1.378 (4)
C12—H12	0.9300	C43—H43	0.9300
C13—C14	1.362 (5)	C44—C45	1.371 (4)
C13—H13	0.9300	C44—C47	1.505 (4)
C14—C15	1.368 (5)	C45—C46	1.379 (4)
C14—H14	0.9300	C45—H45	0.9300
C15—C16	1.370 (5)	C46—H46	0.9300
C15—H15	0.9300	C47—H47A	0.9600
C16—H16	0.9300	C47—H47B	0.9600
C17—C23	1.478 (4)	C47—H47C	0.9600
C17—H17A	0.9700	O51—C51	1.426 (4)
C17—H17B	0.9700	O51—H51	0.8200
N21—C25	1.339 (3)	C51—C52	1.458 (6)
N21—N22	1.370 (3)	C51—H51A	0.9700
N21—H21	0.91 (3)	C51—H51B	0.9700
N22—C23	1.292 (3)	C52—H52A	0.9600
C23—N24	1.373 (3)	C52—H52B	0.9600
N24—C25	1.383 (3)	C52—H52C	0.9600
N24—N25	1.387 (3)	O61—C61	1.429 (11)
C25—S25	1.661 (3)	O61—H61	0.8214
N25—C26	1.284 (3)	C61—C62	1.465 (11)
C26—C34	1.415 (3)	C61—H61A	0.9700
C26—H26	0.9300	C61—H61B	0.9700
O31—N32	1.364 (3)	C62—H62A	0.9600
O31—C35	1.414 (3)	C62—H62B	0.9600
N32—N33	1.299 (3)	C62—H62C	0.9600
N33—C34	1.353 (3)		
			
O1—C1—N1	120.1 (3)	C35—C34—C26	129.2 (2)
O1—C1—C11	122.2 (3)	O35—C35—C34	135.6 (3)
N1—C1—C11	117.8 (3)	O35—C35—O31	121.0 (3)
C1—N1—C17	122.2 (2)	C34—C35—O31	103.4 (2)
C1—N1—H1	119 (2)	C42—C41—C46	121.4 (3)
C17—N1—H1	119 (2)	C42—C41—N33	119.3 (2)
C16—C11—C12	118.3 (3)	C46—C41—N33	119.3 (2)
C16—C11—C1	118.0 (3)	C41—C42—C43	118.3 (3)
C12—C11—C1	123.7 (3)	C41—C42—H42	120.8
C13—C12—C11	120.4 (3)	C43—C42—H42	120.8
C13—C12—H12	119.8	C44—C43—C42	121.9 (3)
C11—C12—H12	119.8	C44—C43—H43	119.0
C14—C13—C12	120.5 (3)	C42—C43—H43	119.0
C14—C13—H13	119.7	C45—C44—C43	117.9 (3)
C12—C13—H13	119.7	C45—C44—C47	121.7 (3)
C13—C14—C15	119.5 (3)	C43—C44—C47	120.4 (3)
C13—C14—H14	120.2	C44—C45—C46	121.6 (3)
C15—C14—H14	120.2	C44—C45—H45	119.2
C14—C15—C16	120.4 (4)	C46—C45—H45	119.2
C14—C15—H15	119.8	C41—C46—C45	118.9 (3)
C16—C15—H15	119.8	C41—C46—H46	120.6
C15—C16—C11	120.8 (3)	C45—C46—H46	120.6
C15—C16—H16	119.6	C44—C47—H47A	109.5
C11—C16—H16	119.6	C44—C47—H47B	109.5
N1—C17—C23	114.9 (2)	H47A—C47—H47B	109.5
N1—C17—H17A	108.6	C44—C47—H47C	109.5
C23—C17—H17A	108.6	H47A—C47—H47C	109.5
N1—C17—H17B	108.6	H47B—C47—H47C	109.5
C23—C17—H17B	108.6	C51—O51—H51	109.5
H17A—C17—H17B	107.5	O51—C51—C52	114.2 (3)
C25—N21—N22	114.5 (2)	O51—C51—H51A	108.7
C25—N21—H21	125.5 (19)	C52—C51—H51A	108.7
N22—N21—H21	120.0 (18)	O51—C51—H51B	108.7
C23—N22—N21	104.3 (2)	C52—C51—H51B	108.7
N22—C23—N24	110.6 (3)	H51A—C51—H51B	107.6
N22—C23—C17	125.6 (3)	C51—C52—H52A	109.5
N24—C23—C17	123.6 (3)	C51—C52—H52B	109.5
C23—N24—C25	109.0 (2)	H52A—C52—H52B	109.5
C23—N24—N25	118.0 (2)	C51—C52—H52C	109.5
C25—N24—N25	132.7 (2)	H52A—C52—H52C	109.5
N21—C25—N24	101.7 (2)	H52B—C52—H52C	109.5
N21—C25—S25	127.9 (2)	C61—O61—H61	97.5
N24—C25—S25	130.4 (2)	O61—C61—C62	110.6 (15)
C26—N25—N24	117.5 (2)	O61—C61—H61A	109.5
N25—C26—C34	117.1 (2)	C62—C61—H61A	109.5
N25—C26—H26	121.4	O61—C61—H61B	109.5
C34—C26—H26	121.4	C62—C61—H61B	109.5
N32—O31—C35	111.2 (2)	H61A—C61—H61B	108.1
N33—N32—O31	104.6 (2)	C61—C62—H62A	109.5
N32—N33—C34	114.9 (2)	C61—C62—H62B	109.5
N32—N33—C41	117.9 (2)	H62A—C62—H62B	109.5
C34—N33—C41	127.2 (2)	C61—C62—H62C	109.5
N33—C34—C35	105.9 (2)	H62A—C62—H62C	109.5
N33—C34—C26	124.7 (2)	H62B—C62—H62C	109.5
			
O1—C1—N1—C17	−9.0 (4)	C25—N24—N25—C26	−27.7 (4)
C11—C1—N1—C17	170.3 (3)	N24—N25—C26—C34	179.0 (2)
O1—C1—C11—C16	−10.8 (4)	C35—O31—N32—N33	−0.9 (3)
N1—C1—C11—C16	169.9 (3)	O31—N32—N33—C34	0.6 (3)
O1—C1—C11—C12	167.1 (3)	O31—N32—N33—C41	−179.5 (2)
N1—C1—C11—C12	−12.2 (4)	N32—N33—C34—C35	−0.1 (3)
C16—C11—C12—C13	−0.7 (4)	C41—N33—C34—C35	−179.9 (3)
C1—C11—C12—C13	−178.7 (3)	N32—N33—C34—C26	−175.8 (2)
C11—C12—C13—C14	−0.1 (5)	C41—N33—C34—C26	4.3 (4)
C12—C13—C14—C15	0.3 (5)	N25—C26—C34—N33	179.9 (2)
C13—C14—C15—C16	0.3 (6)	N25—C26—C34—C35	5.2 (4)
C14—C15—C16—C11	−1.2 (6)	N33—C34—C35—O35	179.7 (4)
C12—C11—C16—C15	1.4 (5)	C26—C34—C35—O35	−4.8 (7)
C1—C11—C16—C15	179.5 (3)	N33—C34—C35—O31	−0.5 (3)
C1—N1—C17—C23	114.4 (3)	C26—C34—C35—O31	175.0 (3)
C25—N21—N22—C23	0.3 (3)	N32—O31—C35—O35	−179.3 (3)
N21—N22—C23—N24	−0.9 (3)	N32—O31—C35—C34	0.9 (3)
N21—N22—C23—C17	−175.0 (3)	N32—N33—C41—C42	52.9 (3)
N1—C17—C23—N22	−124.1 (3)	C34—N33—C41—C42	−127.2 (3)
N1—C17—C23—N24	62.5 (4)	N32—N33—C41—C46	−128.6 (3)
N22—C23—N24—C25	1.1 (3)	C34—N33—C41—C46	51.3 (4)
C17—C23—N24—C25	175.4 (2)	C46—C41—C42—C43	−1.2 (4)
N22—C23—N24—N25	175.0 (2)	N33—C41—C42—C43	177.2 (2)
C17—C23—N24—N25	−10.7 (4)	C41—C42—C43—C44	−0.2 (4)
N22—N21—C25—N24	0.3 (3)	C42—C43—C44—C45	1.4 (4)
N22—N21—C25—S25	−178.8 (2)	C42—C43—C44—C47	−176.8 (3)
C23—N24—C25—N21	−0.8 (3)	C43—C44—C45—C46	−1.1 (4)
N25—N24—C25—N21	−173.5 (2)	C47—C44—C45—C46	177.0 (3)
C23—N24—C25—S25	178.3 (2)	C42—C41—C46—C45	1.4 (4)
N25—N24—C25—S25	5.6 (4)	N33—C41—C46—C45	−177.0 (2)
C23—N24—N25—C26	160.2 (2)	C44—C45—C46—C41	−0.2 (4)

### N-({4-[3-(4-Methylphenyl)-4-sydnonylideneamino]-5-sulfanylidene-1H-1,2,4-triazol-3-yl}methyl)benzamide ethanol monosolvate (II) Hydrogen-bond geometry (Å, º)

**Table:**

*D*—H···*A*	*D*—H	H···*A*	*D*···*A*	*D*—H···*A*
N1—H1···N25	0.83 (3)	2.56 (3)	2.961 (3)	111 (2)
N21—H21···O51^i^	0.91 (3)	1.85 (3)	2.747 (4)	167 (2)
N21—H21···O61^i^	0.91 (3)	1.77 (3)	2.65 (4)	164 (3)
O51—H51···O1	0.82	1.94	2.754 (4)	171
O61—H61···O1	0.82	1.73	2.55 (4)	180
C12—H12···O35	0.93	2.47	3.366 (4)	163

Symmetry code: (i) −*x*+1, −*y*+1, −*z*+1.
